# Ecological predictors of mosquito population and arbovirus transmission synchrony estimates

**DOI:** 10.1093/jme/tjad024

**Published:** 2023-03-25

**Authors:** Joseph R McMillan, Luis Fernando Chaves, Philip M Armstrong

**Affiliations:** Department of Biological Sciences, Texas Tech University, Lubbock, TX, USA; Department of Entomology, The Connecticut Agricultural Experiment Station, New Haven, CT, USA; Department of Environmental and Occupational Health, School of Public Health, Indiana University, Bloomington, IN, USA; Department of Entomology, The Connecticut Agricultural Experiment Station, New Haven, CT, USA

**Keywords:** population synchrony, community ecology, arbovirus, mosquito, public health surveillance

## Abstract

Quantifying synchrony in species population fluctuations and determining its driving factors can inform multiple aspects of ecological and epidemiological research and policy decisions. We examined seasonal mosquito and arbovirus surveillance data collected in Connecticut, United States from 2001 to 2020 to quantify spatial relationships in 19 mosquito species and 7 arboviruses timeseries accounting for environmental factors such as climate and land cover characteristics. We determined that mosquito collections, on average, were significantly correlated up to 10 km though highly variable among the examined species. Few arboviruses displayed any synchrony and significant maximum correlated distances never exceeded 5 km. After accounting for distance, mixed effects models showed that mosquito or arbovirus identity explained more variance in synchrony estimates than climate or land cover factors. Correlated mosquito collections up to 10–20 km suggest that mosquito control operations for nuisance and disease vectors alike must expand treatment zones to regional scales for operations to have population-level impacts. Species identity matters as well, and some mosquito species will require much larger treatment zones than others. The much shorter correlated detection distances for arboviruses reinforce the notion that focal-level processes drive vector-borne pathogen transmission dynamics and risk of spillover into human populations.

## Introduction

Synchrony is defined as the degree of co-variance of spatially disparate populations in time (Loreau and de Mazancourt 2008) and is driven by intrinsic and extrinsic factors, such that coexistence and extinction of species communities can be explained by spatial variability of intrinsic growth rates and responses to environmental forcing (Ranta et al. 2006, Ripa and Ranta 2007, Loreau and de Mazancourt 2008). Synchrony analyses have many applications and have been used in ecological contexts to examine species’ persistence and stability across landscapes (Keitt 2008, Thackeray 2016, Valencia et al. 2020) and in epidemiological contexts to explain pathogen transmission among subpopulations (Althouse et al. 2012) and inform disease intervention strategies (Earn et al. 1998). The underlying processes driving synchrony are the same between ecological and epidemiological contexts; however, the applied implications of synchrony in each discipline differ. Ecologically, asynchrony increases a species’ or community’s resilience and persistence, and interventions that increase asynchrony among populations and/or individuals within a community are an important aspect of conservation efforts, especially for rare or at-risk species/populations (Pardikes et al. 2017). Epidemiologically, synchronous pathogen transmission cycles can overwhelm healthcare systems by affecting multiple locations simultaneously; however, synchrony also increases the likelihood of pathogen extinction since synchronous outbreaks increase the potential for successful control (Earn et al. 1998). Here, we focus on the eco-epidemiological contexts of mosquito species and arbovirus population synchrony by applying synchrony analyses to 20 yr of mosquito and arbovirus surveillance data from Connecticut (CT), United States (U.S.).

Published synchrony analyses typically assess covariance in time series of multiple species or populations as a function of distance, stochasticity (i.e., the Moran Effect, a catch-all term for environmental and/or climatic factors), and inter-species interactions such as competition and predation (Ranta et al. 2006, Pardikes et al. 2017). While inter-species interactions can be important sources of mosquito species population regulation (Ellis et al. 2006, Andreadis and Wolfe 2010, Alto et al. 2012, Chaves 2016), such interactions are seldom investigated in synchrony analyses due to a lack of available data for a target species’ (or guild’s) competitors and/or predators; many species within a species pool may also be considered equivalent if they respond similarly to internal and external forces (Loreau and de Mazancourt 2008), and thus interspecific interactions can be ignored. In the context of mosquitoes and mosquito-borne diseases, prior research has analyzed trends in species collections of *Aedes* and *Culex* spp. mosquitoes along climatic, habitat, and altitudinal gradients (Chaves et al. 2013, Chaves 2017, Chaves et al. 2020), the timing and prevalence of dengue infections in disparate populations (Althouse et al. 2012, Churakov et al. 2019), and the influence of rainfall on malaria epidemics (Chaves et al. 2012a). Theoretical work has also examined the climatic and spatial factors that influence synchrony in mosquitoes. A mathematical model representative of *Culex nigripalpus* Theobald in Florida determined that precipitation patterns can force synchronous population fluctuations (Shaman and Day 2007), while a separate model representative of *Culex pipiens* Linnaeus and *Aedes albopictus* Skuse concluded dispersal rescues populations across expansive and spatially heterogeneous landscapes, which has important implications for the design of local and regional mosquito control programs (Alcalay et al. 2017). While these studies provide evidence that local and regional factors influence synchrony of mosquito species of public health concern, there are no studies that address the generality of synchrony among communities of mosquitoes and the viruses they transmit to humans and wildlife at scales relevant to public health. More direct estimates of synchrony that define spatial patterns of surveillance results (either mosquito collections or arbovirus detections) could provide several applied benefits, such as a correlation decay rate of arbovirus detections among surveillance sites or estimates of treatment area sizes for population-level mosquito control.

The objectives of our research were to i) assess aspects of spatial and temporal synchrony of annual collections for multiple mosquito species and arboviruses of public health concern from longitudinal surveillance data, and ii) identify relationships between estimated synchrony metrics, sampling location distance, habitat, and climate among surveillance sites within a regional surveillance program. We expected some degree of spatial decay in synchrony estimates across all examined mosquitoes and arboviruses. We further hypothesized that features of land cover and climate would be more predictive of synchrony estimates than the Euclidean distances between surveillance sites.

## Materials and Methods

All analyses took place in R V4.1.3 (R Development Core Team 2008) using a variety of packages.

### Surveillance Data

Mosquito and arbovirus data were obtained from ground-level, CO_2_-baited CDC light traps and ground-level hay-lactalbumin infusion-baited gravid traps at 87 surveillance sites in CT from 2001 to 2020. Briefly, sites were sampled by operating traps overnight on a 10-day rotation each summer from June through October, and all female mosquitoes were identified to species using a dichotomous key and tested for nine arboviruses using viral cell culture and RT-PCR techniques (Armstrong et al. 2011). Collection sites were sampled more frequently (1–2 times weekly) for the remainder of a season if mosquitoes tested positive for an arbovirus of primary concern; while the CT program generally tests for abroviruses, the primary abroviruses of concern are all zoonotic with West Nile virus and eastern equine encephalitis virus being the most important to public health. The CT surveillance program has expanded from 36 sites in 1997 to 108 sites in operation today; major expansions in the network occurred in 2000 following the introduction of West Nile virus and in 2020 after the regional expansion of EEEV. Because of the multiple trap types, unequal sampling schemes employed, and differing time scales of sampling at each site, we implemented a series of data restrictions. First, we limited all community-level analyses to light trap data since light traps are among the least biased sampling methodologies for mosquitoes (Silver 2008). Second, we aggregated collections to site-specific annual totals which were then corrected for annual sampling effort (i.e., total divided by the number of times a site was sampled in a season). Third, we de-trended each species-site time series by analyzing residuals from a linear regression of the corrected annual collections. We chose this approach for two reasons: i) prior research using this dataset identified a positive annual increase in mosquito collections for multiple species (Petruff et al. 2020), and ii) each species-site time series had only twenty observations which is too few for more sophisticated detrending methods (Shumway 2017). Fourth, we limited our analyses to 87 sites that have been in continuous operation since 2001. Our final restriction was to examine only species that are endemic to CT and that lay multiple clutches of eggs throughout the summer months. These restrictions avoided the inclusion of single-generation species that develop as larvae before the beginning of a surveillance season, invasive species currently undergoing population expansions (Armstrong et al. 2017), and the inclusion of rare species (Petruff et al. 2020). Nineteen mosquito (Diptera: Culicidae) species out of the 54 total species detected in CT since 2001 remained after implementing this restriction: *Aedes cantator* Coquillett*, Aedes cinereus* Meigen*, Aedes sollicitans* Walker*, Aedes taeniorhynchus* Wiedemann*, Aedes triseriatus* Say*, Aedes trivittatus* Coquillett*, Aedes vexans* Miegen*, Anopheles crucians* Wiedemann*, Anopheles punctipennis* Say*, Anopheles quadrimaculatus* Say*, Anopheles walker* Theobald*, Culiseta melanura* Coquillett*, Culiseta morsitans* Theobald*, Culex pipiens* Linnaeus*, Culex restuans* Theobald*, Culex salinarius* Coquillett*, Culex territans* Walker*, Psorophora ferox* Humboldt, and *Uranotaenia sapphirine* Osten Sacken. Detailed natural history information regarding these species can be found in prior community ecology work using the CT surveillance program data (McMillan et al. 2020). For arboviruses, we restricted our analyses to 7 of 9 endemic arboviruses, including Cache Valley virus (CVV), Eastern equine encephalitis virus (EEEV), Highlands J virus (HJV), Jamestown Canyon virus (JCV), Potosi virus (PTV), Trivittatus virus (TVTV), and West Nile virus (WNV). Excluded arboviruses included La Crosse virus and Flanders virus since these arboviruses are rarely detected. We confirmed time series were successfully detrended by determining there were no significant lag effects, determined using partial autocorrelations using the *pacf* function in the ‘tseries’ R package (Trapletti et al. 2022).

### Land Cover and Climate Data

Monthly temperature and precipitation estimates from 2001 to 2020 were obtained from PRISM (https://prism.oregonstate.edu/) [8] (Daly et al. 2008) by extracting data to the specific surveillance site coordinates using the Data Explorer tool (https://prism.oregonstate.edu/explorer/). PRISM uses a default 4 km grid system, meaning climate values were assigned to points based on the grid in which they are located. Since the CT surveillance program only operates traps from June to October, we extracted monthly values for each site that aligned with the mosquito collection period. Land cover data were obtained from UCONN CLEAR (https://clear.uconn.edu/projects/landscape/index.htm). Briefly, this site hosts data on twelve land cover classes for Long Island Sound from 1985 to 2015 (at intervals of 5 yr). Shapefiles of 2015 land cover estimates were obtained and then processed in ArcMap (ESRI) using a combination of clipping and spatial joins to produce a.csv of the acreage of each land cover type within ~500 m of a site; percent land cover per trap buffer was then calculated in R. Published research has shown that overall land cover patterns have changed little in CT (Arnold et al. 2020), so we did not include any other estimates of land cover concurrent with the surveillance period.

We converted all land cover and climate data into indices of (dis)similarity using the *vegdist* function in the R package ‘vegan’ (Oksanen et al. 2013). This method condenses multivariate data to a value between 0 and 1 with values of 0 indicating full similarity and values of 1 indicating full dissimilarity. Our rationale was that indices of similarity, rather than raw, observed values of habitat and climate, provide a singular unit of ‘distance’ akin to Euclidean distance; therefore, (dis)similarity indices allow for centering in subsequent analyses. For climate similarities, we generated similarity indices for precipitation and temperature independently as well as a single climate index which accounted for both temperature and precipitation monthly values.

### Community Synchrony

We utilized functions available in the R package ‘synchrony’ (Gouhier and Guichard 2014) to quantify metrics of spatial synchrony among species and arboviruses. We first determined the degree to which mosquito and arbovirus communities are synchronous within a site using the *community.sync* function. This function generates a value between 1/S and 1 with S equal to the number of species in the community: values of 1 indicate a complete correlation of time series fluctuations while values of 1/*S* indicate uncorrelated fluctuations. We then compared community synchrony estimates to individual land cover classes using simple Pearson correlation tests to assess if any land cover variables were associated with community synchrony estimates. We then tested for spatial autocorrelation among community synchrony estimates using a global Moran’s I test function available in the ‘ade4’ R package (Dray and Dufour 2007). Briefly, the Moran’s I test measures the spatial correlation of the target metric(s) based on the location of all sites; significantly positive correlations indicate positive spatial autocorrelation (i.e., values are similar when locations are closer) while significantly negative spatial autocorrelations indicate an inverse spatial correlation (i.e., values are similar when farther apart). The ‘ade4’ method requires that the collection locations be converted to distance matrixes using a distance function, i.e., *dist* in R.

### Population Synchrony

Species-specific synchrony investigations included the development of correlograms for each species as a function of Euclidean distance between sites using the *vario* and *plot.vario* functions in the ‘synchrony’ package (Gouhier and Guichard 2014). These functions assess correlations in collections as a function of geographic distance using all species-site-specific time series. This function was also used to compare process-specific (i.e., environmental features, temperature, etc.) decay functions; these assessments were qualitative in nature, meaning we visually compared patterns of mosquito or arbovirus species correlograms to patterns of temperature and precipitation correlograms. Due to the CT surveillance program’s partial focus on WNV surveillance, we repeated these species-specific investigations for *Cx. pipiens* and *Cx. restuans* collections and WNV detections in gravid traps. All gravid trap data were detrended following the protocols described above for light traps, and all synchrony methods remained the same for gravid trap data.

### Predicting Population Synchrony

The above analyses identified general patterns of population synchrony as a function of either location (community synchrony estimates) or distance (species-specific synchrony estimates); they did not identify the underlying mechanisms that determine synchrony. To better isolate the influence of distance, habitat, and climate on synchrony estimates, we first quantified a metric of phase synchrony of each species’ annual time series for all pairwise combinations of sites using the *phase.sync* function in the ‘synchrony’ package (Gouhier and Guichard 2014). Briefly, this function estimates phase locking of maxima and minima shared between two time series and generates values between 0 (no phase synchrony) and 1 (complete phase locking), with phase locking defined as maxima and minima occurring in complete tandem. In our analyses, we parameterized *phase.sync* functions using mod=1, method=‘fft’, and nrand=100. Phase synchrony estimates were then examined for spatial patterns using the *mantel* function in the ‘vegan’ package. We chose this function because it allows for the exclusion of ‘NA’ values which were generated by our phase synchrony methods. Species and viruses with evidence of spatial patterns of phase synchrony, as a function of the Euclidean distance between the time series’ sites, were excluded from any further investigations.

We then used generalized linear mixed effects models (GLMMs) to determine how distance, climate, and land cover explain variance in mosquito and arbovirus metrics of phase synchrony. We first defined a null model containing log-transformed phase synchrony estimates (adding 1) as the response variable, distance between sites as a fixed effect, species identity as a random effect, and a Gaussian error distribution. We used the log transformation to impose a negative exponential fit to the response ~ distance relationship, which prior research has shown to be the best fitting model of similarity metrics based on distance (Gómez-Rodríguez and Baselga 2018); the +1 was to prevent issues with logging zero values. Model selection followed a user-defined forward selection approach in which single terms were assessed by comparing changes in the Akaike Information Criteria to the null model (ΔAIC_null_). Terms displaying the greatest improvement in ΔAIC_null_ > 2 were sequentially evaluated in a multi-term model until ΔAIC was maximized using only additive terms (i.e., ΔAIC_add_). Once the best-fitting additive model was found, we then assessed interactions between terms, only keeping interactions that improved ΔAIC_add_ > 2. No three-way interactions were assessed due to the difficulty of interpreting the biological meaning of such terms (Bolker et al. 2009). All GLMMs were fit using functions available in the ‘glmmTMB’ package (Brooks et al. 2017). Final candidate model predictions and random effects were then assessed using a combination of functions available in the ‘ggplot2’, ‘ggeffects’, and ‘tidyverse’ packages (Wickham et al. 2016, Wickham et al. 2019, Lüdecke 2018).

## Results

### Community Synchrony

**Fig. 1.**
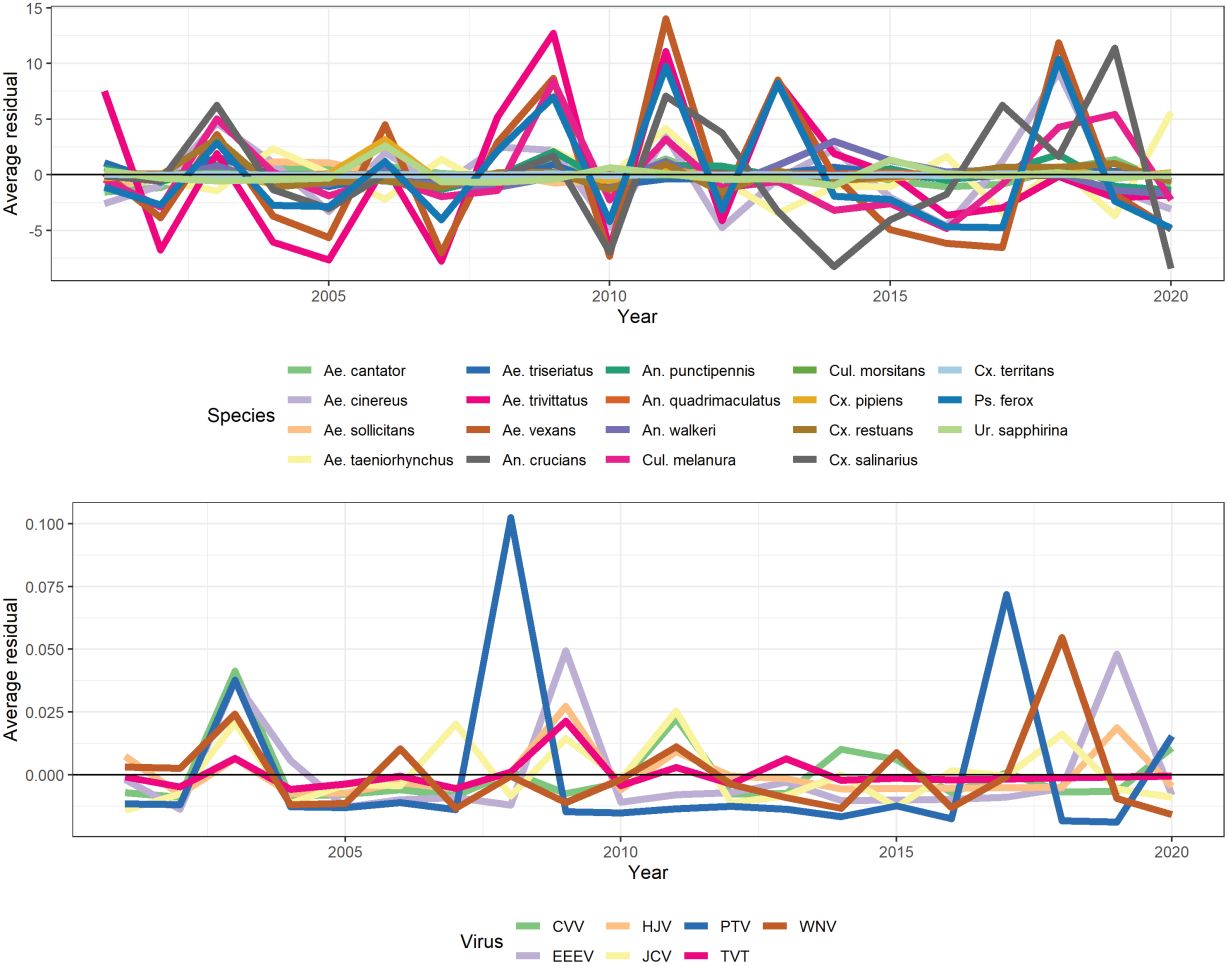
Detrended average (i.e., state-level) annual mosquito collections (top row) and arbovirus detections (bottom row) in Connecticut, United States, from June to October from to 2020.

**Fig. 2.**
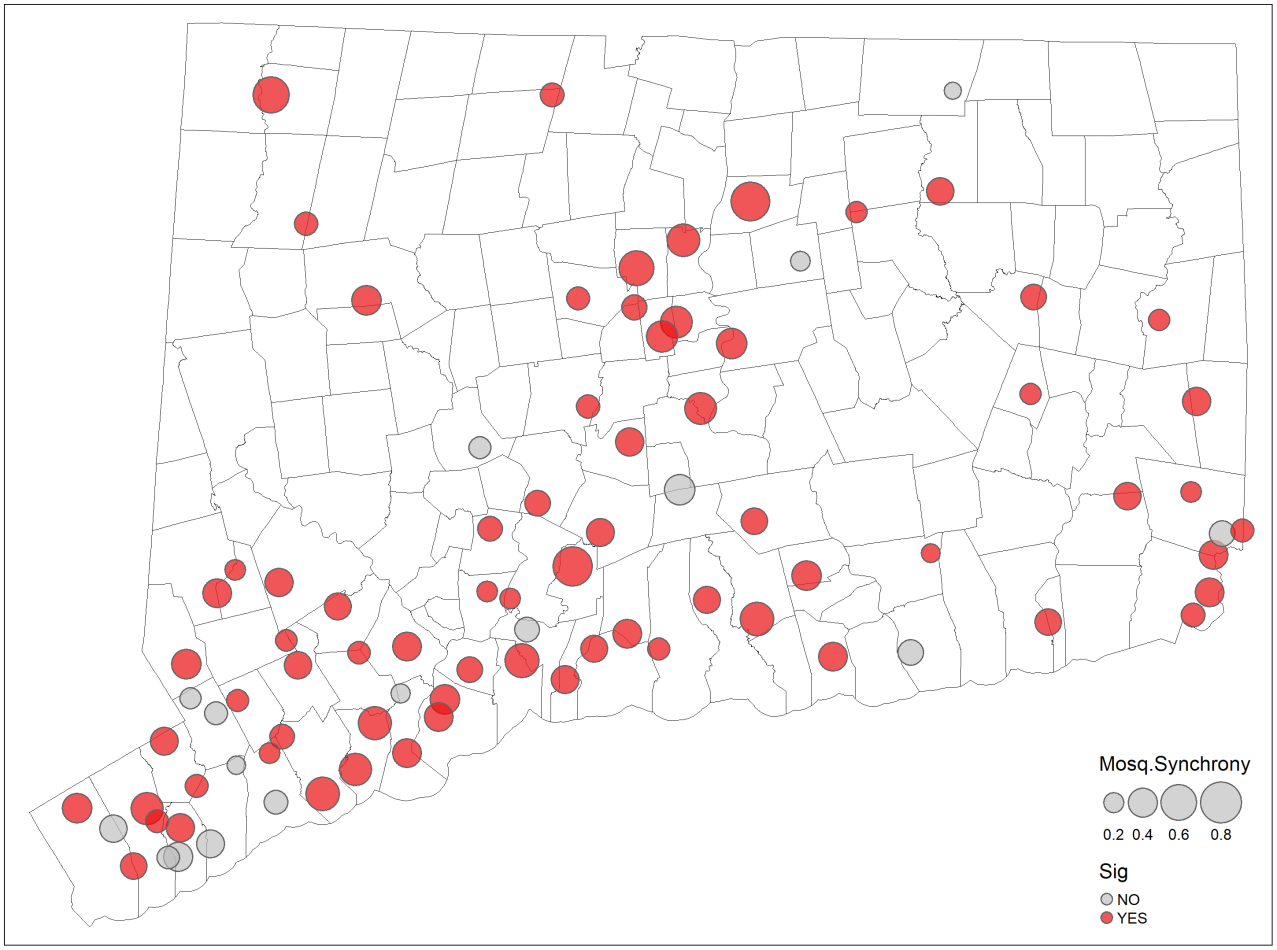
Site-level mosquito community synchrony estimates based on annual collections of 19 mosquito species in Connecticut, United States. Black lines indicate the primary local political units in CT (towns). The size of the point corresponds to the magnitude of the synchrony estimate; the color indicates significance at the *P* < 0.05 level (See online version for color figure).

The objective was to estimate the level of interspecific synchrony of mosquito or arbovirus collections at individual sites and evaluate any relationships between community synchrony estimates and land cover. Detrending approaches successfully removed residual temporal correlations within site-specific time series for each mosquito and arbovirus species (i.e., there were no significant time-lags or increasing/decreasing trends away from 0). However, visual assessment of the average (i.e., state-level) time series indicated that many mosquito species collections rise and fall together while there were no apparent trends in average arbovirus collections (Fig. 1). Community synchrony estimates supported our visual assessment of average mosquito collections: 80.5% of community synchrony estimates were significantly greater than 1/*S* (Fig. 2). There was no evidence for global spatial autocorrelation of mosquito community synchrony estimates, and there were no significant associations between mosquito community synchrony estimates and any land cover type. Arbovirus community synchrony estimates were also on average greater than 1/*S*; estimates were exceedingly high (>0.8 with a mean of 0.93) while no estimates were determined to be significant at *P* < 0.05. We determined this was due to the commonality of *not* detecting any arboviruses at any given site in any given year. No further examinations of arbovirus community synchrony were pursued beyond comparing site-specific mosquito and arbovirus community metrics using a correlation test, which did not identify any relationship between the two metrics.

### Population Synchrony

**Fig. 3.**
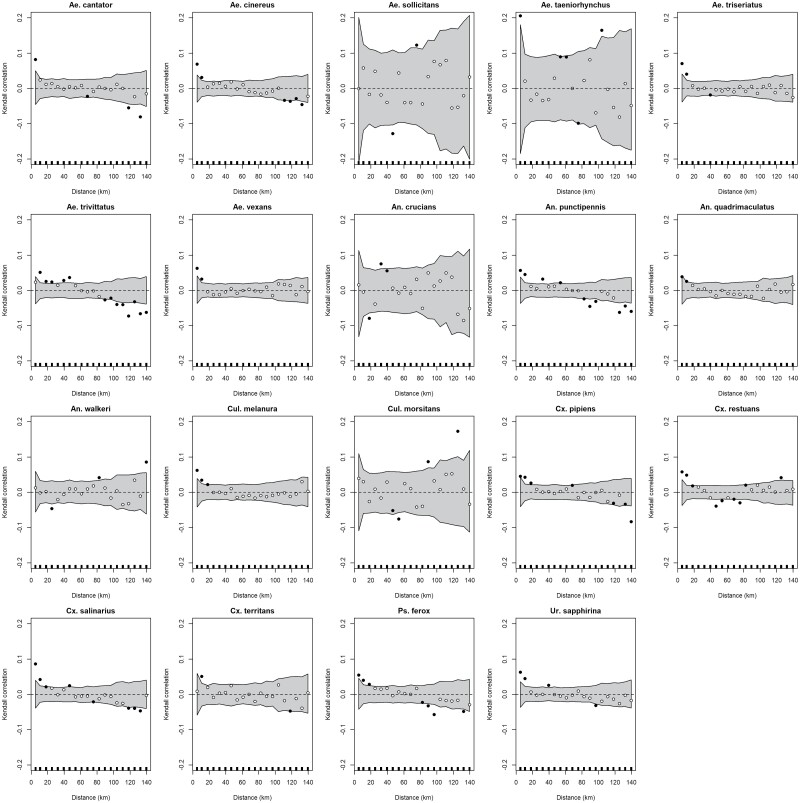
Spatial correlograms for 19 endemic mosquito species in Connecticut, United States. Each plot is centered, meaning that the regional mean is subtracted from each value. Points represent the estimated correlation per distance band (black: significant at *P* < 0.05; white: not significant) while the gray-shaded region represents 95% CI of the mean correlation.

**Fig. 4.**
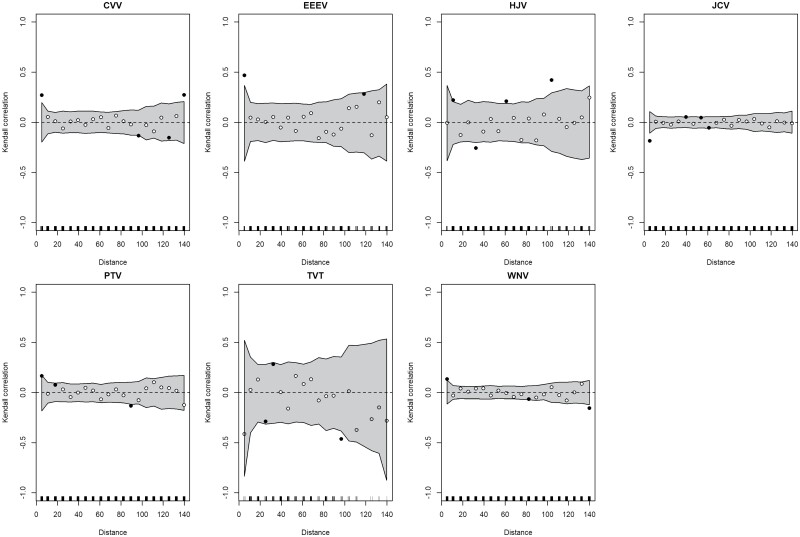
Spatial correlograms for 7 endemic arboviruses detected in Connecticut, United States. Each plot is centered, meaning the regional mean is subtracted from each value. Points represent the estimated correlation per distance band (black: significant at *P* < 0.05; white: not significant) while the gray shaded region represents that 95%CI of the mean correlation.

The objective was to estimate the spatial synchrony of mosquito and arbovirus-specific collections between each site in the CT surveillance program. All spatial correlograms evaluated the correlation of collections at twenty distance bands between 0 and 140 km. Fourteen mosquito species displayed a pattern of significantly correlated collections up to at least five kilometers, and the average maximum significantly correlated distance was ~ 10 km (Fig. 3). A linear fit to the correlation-distance relationships best explained the spatial decay in mosquito species collection correlations, i.e., collections tended to be positively correlated at shorter distances and negatively correlated at farther distances (Fig. 3). Re-examining *Culex* spp. collections in gravid traps extended the estimated maximum distance of significantly correlated collections from ~18 km to 40 km for *Cx. pipiens* (Supplementary Fig. 1). Maximum correlated detection distances for arboviruses were much shorter than for mosquitoes, and five arboviruses displayed a significant collection correlation up to 5 km: CVV, EEEV, PTV, and WNV were all positive while JCV was negative (Fig. 4). Re-examining WNV detections in gravid traps did not increase in the maximum distance of significantly correlated detections (Supplementary Fig. 1). Additionally, no decay function available in *vario* function explained patterns of any arbovirus detections in light or gravid traps based on distance.

### Predicting Population Synchrony

The objective was to predict estimates of mosquito and arbovirus-specific synchrony as a function of habitat, climate, species or arbovirus identity, and distance between sites. Our initial Mantel tests of phase synchrony estimates identified a significant distance effect in a single species, *Cx. salinarius*, and this species was excluded from further analyses. All examined collections from gravid traps (*Cx. pipiens, Cx. restuans*, and WNV isolations) showed a significant distance effect, and GLMMs of gravid trap data were not performed.

**Fig. 5.**
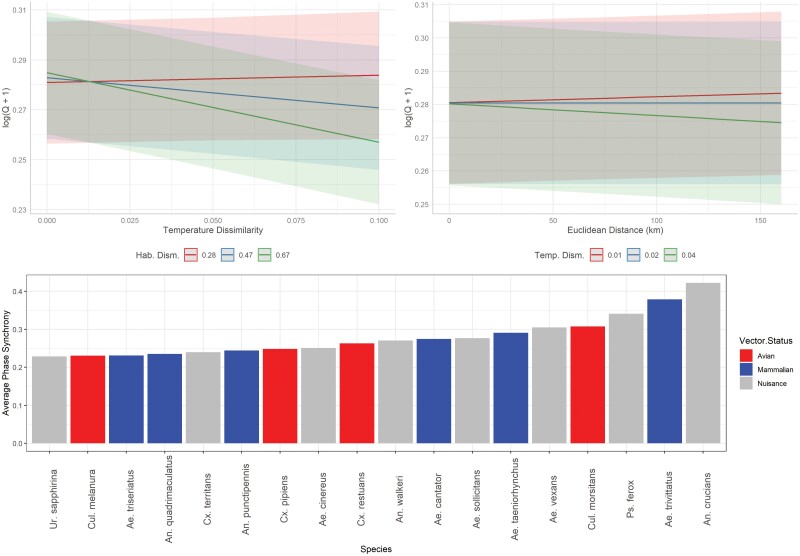
Fixed effect predictions of pairwise measures of time series phase synchrony for 18 mosquito species collected in Connecticut, United States. Predictions generated from a generalized linear mixed-effects model with log + 1 transformed phase synchrony estimates, Euclidean distance, temperature similarity, habitat similarity, a temperature/habitat and a distance/temperature interaction as fixed effects, and species identity as a random effect.

**Fig. 6.**
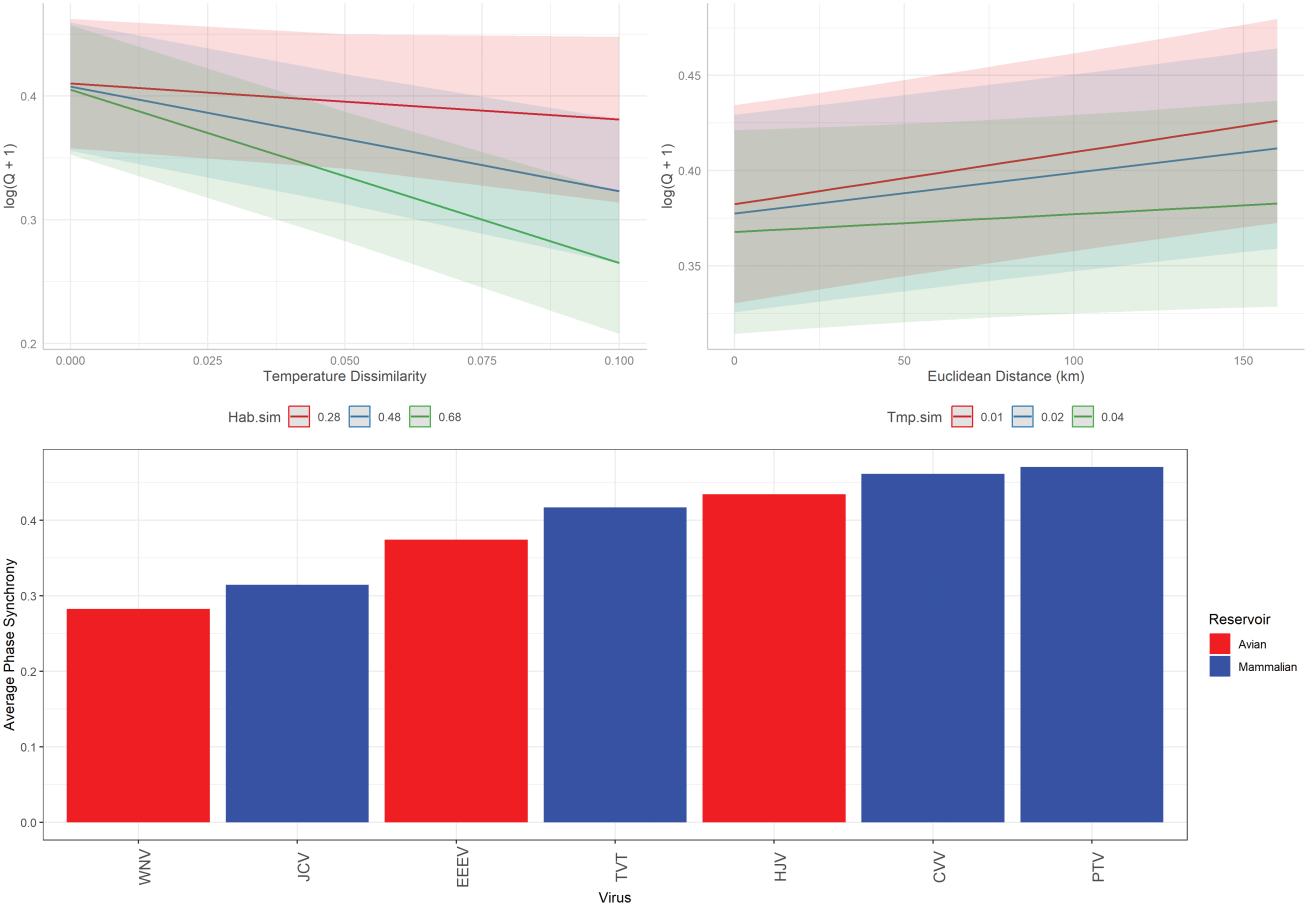
Fixed-effects predictions of pairwise measures of time-series phase synchrony for 6 arbovirus detections in Connecticut, United States. Predictions generated from a generalized linear mixed-effects model with log + 1 transformed phase synchrony estimates, Euclidean distance, temperature similarity, habitat similarity, and a temperature/ habitat interaction as fixed effects, and arbovirus identity as a random effect.

Our model selection approach produced a final model for mosquito and arbovirus phase synchrony that included the following terms: distance, temperature, and habitat dissimilarity indexes, an interaction between temperature and habitat, and an interaction between temperature and distance (mosquito species only) as fixed effects, and species/arbovirus ID as a random effect (Table 1). Based on *F*-values of the fixed effects, temperature dissimilarity explained more variation in mosquito species synchrony while habitat dissimilarity explained more variation in arbovirus synchrony (Table 1, Figs. 5 and 6). The next most important fixed effect variable for mosquito synchrony was the temperature by habitat interaction; for arboviruses, temperature was the second-ranked fixed effect (Table 1). *F*-values did not directly correlate with coefficient p-values generated by *glmmTMB*; since each additive term improved ΔAIC_null_ > 2, this is likely due to the presence of the interaction terms in each model. For both mosquito and arbovirus GLMMs, dissimilarity indexes of precipitation or climate (which accounted for temperature and precipitation together) did not improve AIC beyond AIC_null_.

**Table 1.** ANOVA-style table generated from a generalized linear mixed-effects model with log + 1 transformed phase synchrony estimates, Euclidean distance, temperature similarity, habitat similarity, and a temperature/ habitat interaction as fixed effects, and mosquito species or arbovirus identity as a random effect. GLMMs were analyzed using the R package ‘glmmTMB’ and ANOVA values were generated using the *aov* function in base R

Variable	Range	Mosquito Species			Arbovirus		
		MSE	*F*-value	*P*-value	MSE	*F*-value	*P*-value
Distance (km)	0–150 km	0.161	9.94	0.09	0.066	1.86	0.0001
Temperature Dissimilarity	0–0.1	1.677	103.52	2.0e-5	1.288	36.6	0.12
Habitat Dissimilarity	0–1	0.119	7.35	0.0064	3.297	93.7	0.76
Temperature * Habitat interaction	NA	0.789	48.7	3.1e-9	0.798	22.7	2.0e-6
Temperature * Distance interaction	NA	0.088	5.47	0.02	–	–	–
*Random*: Species	Mosquito: df = 17 Arbovirus: df = 6	13.758	854.5	–	10.700	304.00	–
*Residual*	Mosquito: df = 107,259 Arbovirus: df = 10,600	0.0162	–	–	0.0352	–	–

In general, phase synchrony estimates shared a negative relationship with temperature and habitat (dis)similarity: as sites became less similar, phase synchrony estimates decreased (Figs. 5 and 6). Mosquito species phase synchrony estimates remained flat as a function of distance while arbovirus estimates increased with distance (Figs. 5 and 6). However, the AIC_null_ model for arboviruses determined that distance shared a negative, though not significant, association with phase synchrony, likely indicating the distance relationship in the final AIC_int_ model is due to the included additive and interaction terms. Despite these distance, temperature, and habitat patterns, the overall predictive capabilities of these variables were minimal compared to the predictive capabilities of species identity (Figs. 5 and 6). Changing mosquito species or arbovirus identity could result in >50% change in predicted synchrony estimates (Figs. 5 and 6). Checking GLMM random effects, there was no clear pattern of synchrony estimates among mosquito species based on vector status or life history characteristics; a similar lack of patterns was observed among the arboviruses (Supplementary Figs. 2 and 3). Additionally, no random intercept estimate for mosquito species or arboviruses was considered significant from another as all confidence intervals of the estimates overlapped (Supplementary Figs. 2 and 3).

## Discussion

Identifying patterns of mosquito and arbovirus population synchrony in space can provide valuable information on spatial targets for public health surveillance and communication programs as well as mosquito control operations. Our mosquito species community synchrony results show that within any given site in the CT surveillance program, mosquito species fluctuate synchronously with no identifiable influence of a specific land cover type. This result is not unexpected since multiple land cover types are associated with mosquito community structure (McMillan et al. 2020) and site similarity indices ranged from completely dissimilar to completely similar. We also found that arbovirus communities were highly synchronous, though this is likely due to the overall low probability of detecting any arbovirus rather than the occurrence of synchronous transmission cycles. We found no correlation between site-specific mosquito community and arbovirus community synchrony estimates, which reinforces prior research showing that few, if any, factors that explain mosquito community structure also explain arbovirus community structure (McMillan et al. 2020). However, the lack of a correlation could be because arbovirus transmission depends on multiple host-vector-pathogen interactions (Reisen 2010), and host-pathogen data is critically lacking in surveillance programs of zoonotic, vector-borne pathogens. Without such data, there are limits to what can be inferred from detected mosquito arbovirus infections in space and time. Our data aggregation to annual time scales could also have hindered our ability to detect meaningful patterns in arbovirus community synchrony. Each arbovirus has a distinct transmission period, with JCV typically considered a late-spring, early-summer arbovirus, WNV as a mid-, late-summer arbovirus, and CVV, EEEV, and HJV are typically late-summer, early fall arboviruses (Andreadis et al. 2008, 2014, McMillan et al. 2020, Armstrong and Andreadis 2022); TVTV can be detected at any point during the summer. Thus, seasonal rather than interannual investigations may be more important to understanding the community synchrony of arboviruses. However, because sites in the CAES surveillance program can be prioritized based on arbovirus results during any given season, such finer temporal scale analyses of arboviruses are more appropriate for specific vector-virus studies, which were outside the scope of this project.

We also found that while significant community synchrony estimates generally occurred across sites, there was no evidence of spatial autocorrelation among estimates. This indicates that regularly sampled, individual trap sites effectively capture members of a site’s community, and if the population of one species is elevated at a particular site, it is likely that other species’ populations are also elevated. This result however could be due to our restrictions to endemic, multi-generational, summer mosquitoes. Though these nineteen species may vary in their specific life histories, being summer species, their populations all likely similarly react to regional climatic processes. Additionally, because our analyses utilized light traps, this suggests that all captured adults were host seeking, and the impacts of precipitation, temperature, and humidity all likely influence host-seeking behaviors across species. The lack of a correlation between community synchrony estimates and land cover variables as well as a lack of spatial autocorrelation could also be due to the size of the examined program. Connecticut is a small U.S. state, and the CT surveillance program tends to oversample the two dominant land cover types: developed and forested habitats (McMillan et al. 2020). Analyses that combine surveillance data sets across larger spatial distances and include a greater variety of habitats may find stronger relationships among traps as a function of habitat and distance. Study design can also influence population-level inference, and stratified-random trapping designs guided by historical knowledge of mosquito dynamics out-perform random and transect-level designs (Reisen and Lothrop 1999). While the spatial coverage and design of the CT program may limit further investigations into the general ecology of mosquito community dynamics, the CT program is first and foremost a public health service, so a biased sample design is to be expected. We note here that the lack of a spatial pattern of community or species-specific population synchrony estimates within the network emphasizes the importance of continued monitoring at each site – all sites are somewhat unique and require regular sampling to understand the risk of mosquito bites to nearby residents.

We also found general patterns of population synchrony among individual mosquito species and arboviruses. Our species-specific analyses showed that most mosquito species collections are significantly correlated up to 10 km, with some species collections being significantly correlated up to 20 or 40 km. Importantly, there was no easily identifiable pattern of correlated collection distances based on vector status or natural history. For example, *Ps. ferox* and *Ae. trivittatus* had the highest significantly correlated collection distances and are each flood water species. However, the dynamics of these species’ response to precipitation is different. *Psorophora ferox* is considered a mass hatch species and egg hatching occurs in response to flooding of grassy habitats (Darsie and Ward 1981). *Ae. trivittatus* is a more localized, nuisance species, with eggs typically deposited in the grassy margins of streams and/or ponds, though oviposition choices can expand in the summer months (Darsie and Ward 1981). As another example, *Cs. melanura* and *Cx. pipiens* are each vector of avian arboviruses and displayed similar correlated collection distances. However, each of these species differs widely in preferred habitat (*Cs. melanura* larvae are typically found in bogs and forested wetlands while *Cx. pipens* larvae are found in urban centers) (McMillan et al. 2020). It is also worth noting the observed significant correlated collection distances were sensitive to the method of collection, and *Cx. pipiens* correlation distances doubled when analyzed using gravid traps.

There were fewer spatial patterns of arbovirus collections, with the average correlated collection never exceeding 5 km. Arbovirus identity, however, did explain a large amount of variance in phase synchrony estimates. Due to the fewer number of viruses, identifying shared patterns in synchrony estimates is limited. Overall, the mammalian arboviruses displayed a higher degree of phase synchrony than the avian arboviruses. Average phase synchrony estimates for CVV, PTV, and TVT were ~50% higher than WNV and ~30% higher than EEEV (WNV and EEEV are the two primary arboviruses of public health concerns in CT). This could be due to several factors: prior research has shown that mammalian arboviruses are transmitted by multiple vector species, are widely dispersed in CT, and all share white-tailed deer as the primary reservoir host (Andreadis et al. 2014, McMillan et al. 2020). JCV displayed the lowest synchrony estimates of the mammalian arboviruses, and this could be because this virus is typically transmitted by single-generation, early summer mosquito species (Andreadis et al. 2008, McMillan et al. 2020); many of these species were excluded from the analysis. Among the three avian arboviruses, synchrony estimates were similar for EEEV and HJV. These two viruses share the same primary vector (*Cs. melanura*) and detection of these viruses aggregates in the same hardwood, forested wetland sites in the eastern portion of CT (McMillan et al. 2020). Detection of these arboviruses is typified by long periods of minimal detection followed by seasonal epizootics and epidemics (Armstrong and Andreadis 2022); synchrony estimates may reflect such aggregated and intense transmission cycles. Synchrony estimates for WNV were the lowest, which may be due to light traps being inefficient for WNV detection (Yee et al. 2023).

We found a strong pattern of spatial decay in our indices of temperature and precipitation similarity among sites (Supplementary Fig. 4); our GLMMs also identified temperature as an important predictor of synchrony for mosquitoes and arboviruses. Prior research has shown the importance of precipitation to larval population dynamics (Shaman et al. 2003, Shaman and Day 2007, Koval and Vazquez-Prokopec 2018) and temperature on the survival of adults (Mordecai et al. 2019). While we did not find an effect of precipitation on synchrony estimates, this could be because our analyses did not analyze larval data which would have been more sensitive to precipitation. Prior research has also shown that the factors that shape larval community dynamics occur at much shorter spatial distances than examined in this report, likely because the interplay between habitat type and rainfall manifest at very fine spatial scales (Ellis et al. 2006, Paradise et al. 2008). Temperature and precipitation are also associated with trap counts – higher temperatures are associated with higher collections while high precipitation events lead to fewer collections. Since variation in climate dissimilarity indices was minimal (temperature dissimilarity among sites only varied between 0 and 0.12), the similar experience of temperature across sites could further explain why temperature was the most predictive of synchrony estimates among the examined fixed effects despite explaining much of the variance in synchrony estimates. Given the complexity of climate impacts on larval and adult mosquitoes (Chaves et al. 2012b), future studies should examine the similarities and differences in synchrony among these two life stages.

## Conclusion

Ecologically, species identity, rather habitat and climate, explains the most variance in synchrony estimates among mosquito and arbovirus species. This was an unexpected result, and it suggests that species-specific intrinsic processes, rather than stochastic processes, drive patterns of population synchrony at the spatial and temporal scales examined. Follow-up studies should confirm or refute these results using more thorough examinations of how species-specific natural history characteristics, as well as metrics of community composition (i.e., richness/evenness), explain synchrony and other population dynamics. Epidemiologically, the results of our study can inform the implementation of mosquito control operations and public health messaging systems for mosquito-borne diseases in the northeast US. We suggest that mosquito intervention techniques and protocols scale to the target species, and all current programs likely need to expand spatial treatment zones to have population-level impacts on mosquito communities. The overall lack of synchrony patterns among arboviruses suggests a high degree of resilience in arbovirus communities and populations, and interventions to prevent arbovirus spillover to humans may need to intensely focus on high-risk foci as they are detected. Generally, asynchronous arbovirus detections also suggest that other metrics of arbovirus risk are likely better suited to communicating mosquito-borne disease risk to the public, such as highlighting areas and times of current and historic activity, reinforcing and supporting mosquito control and mosquito-borne disease educational campaigns in high-risk demographics, and developing new techniques which project arboviral risk into unsampled spaces.

## Supplementary Material

tjad024_suppl_Supplementary_Material

## Data Availability

All data are archived in Mendeley Data using the following doi: 10.17632/6d4h6x87nb.1.
