# Supplementary material for: Ecological predictors of mosquito population and arbovirus transmission synchrony estimates
Source: J Med Entomol. 2023 Mar 25;60(3):564–74. doi: 10.1093/jme/tjad024 (PMC10179454; doi:10.1093/jme/tjad024)
Supplement: tjad024_suppl_Supplementary_Material [file tjad024_suppl_supplementary_material.docx]

**Supporting Information Figures for:**

**Title:** Ecological predictors of mosquito population and arbovirus transmission synchrony estimates

**Authors:** Joseph R. McMillan^1,3^, Luis Fernando Chaves^2^, Philip M. Armstrong^3^

**Author affiliations:**

^1^Department of Biological Sciences, Texas Tech University, Lubbock, TX

^2^Department of Environmental and Occupational Health, School of Public Health, University of Indiana, Bloomington, IN

^3^Department of Entomology, The Connecticut Agricultural Experiment Station, New Haven, CT

**Corresponding Author:**

Joseph R. McMillan

Biology Building, Rm 212

2901 Main Street

Lubbock, TX 79404

[josmcmil@ttu.edu](mailto:josmcmil@ttu.edu)

ORCID 0000-0002-6909-950x


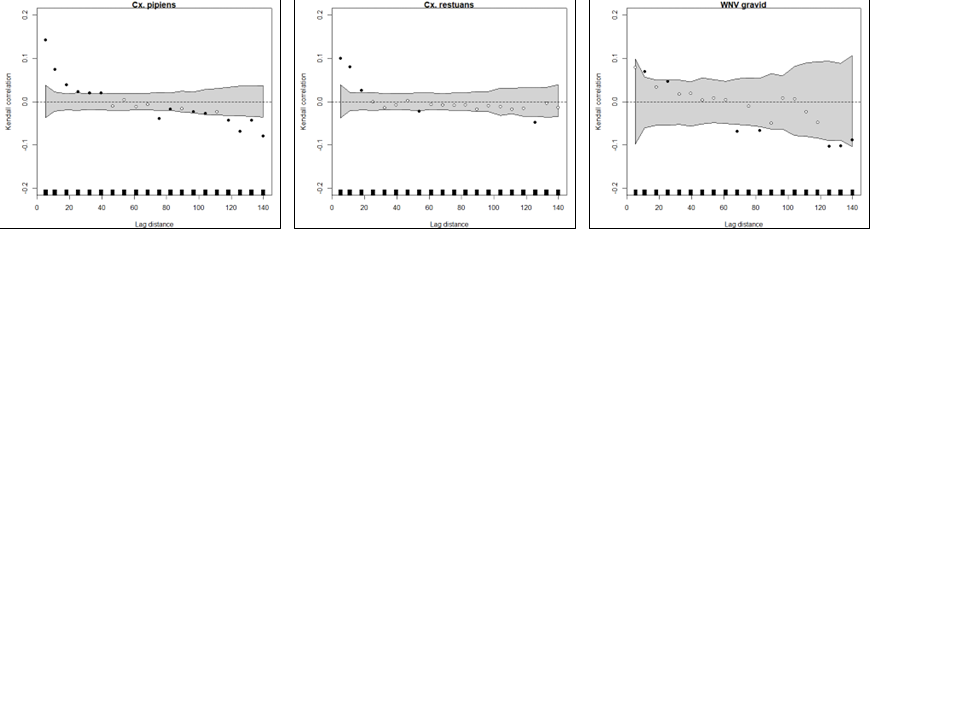


**SI Figure1**. Spatial correlograms for *Culex pipiens*, *Culex restuans*, and West Nile virus collections using CDC ground-level gravid traps set at 87 sites in Connecticut, United States from June to October from 2001 – 2020. Each plot is centered, meaning the regional mean is subtracted from each value. Points represent the estimated correlation per distance band (black: significant at p < 0.05; white: not significant) while the grey shaded region represents that 95%CI of the mean correlation.


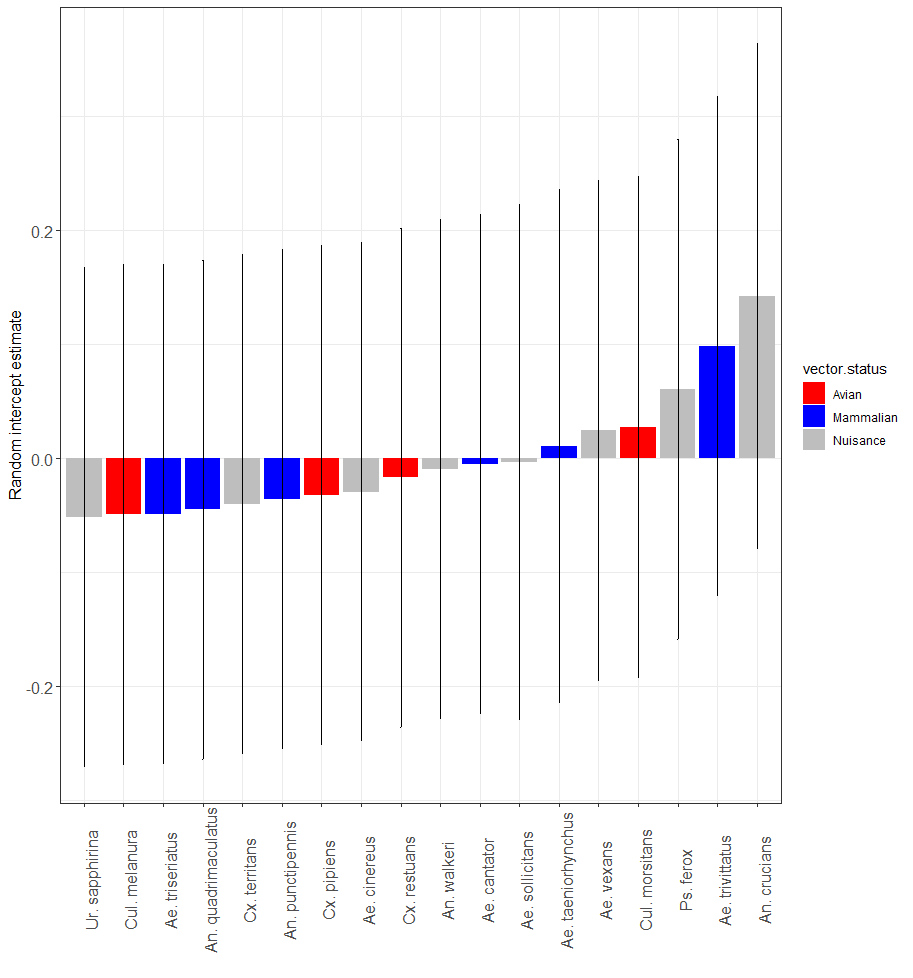


**SI Figure 2**. Mosquito species-specific random intercept estimates of pairwise measures of time series phase synchrony for 18 mosquito species collected in CO_2_-baited, ground-level light traps set at 87 sites in Connecticut, United States from June to October from 2001 – 2020. Predictions generated from a generalized linear mixed effect model with log+1.01 transformed phase synchrony estimates, Euclidean distance, temperature similarity, habitat similarity, and a temperature/ habitat interaction as fixed effects, and mosquito species identity and site name as crossed random effects.


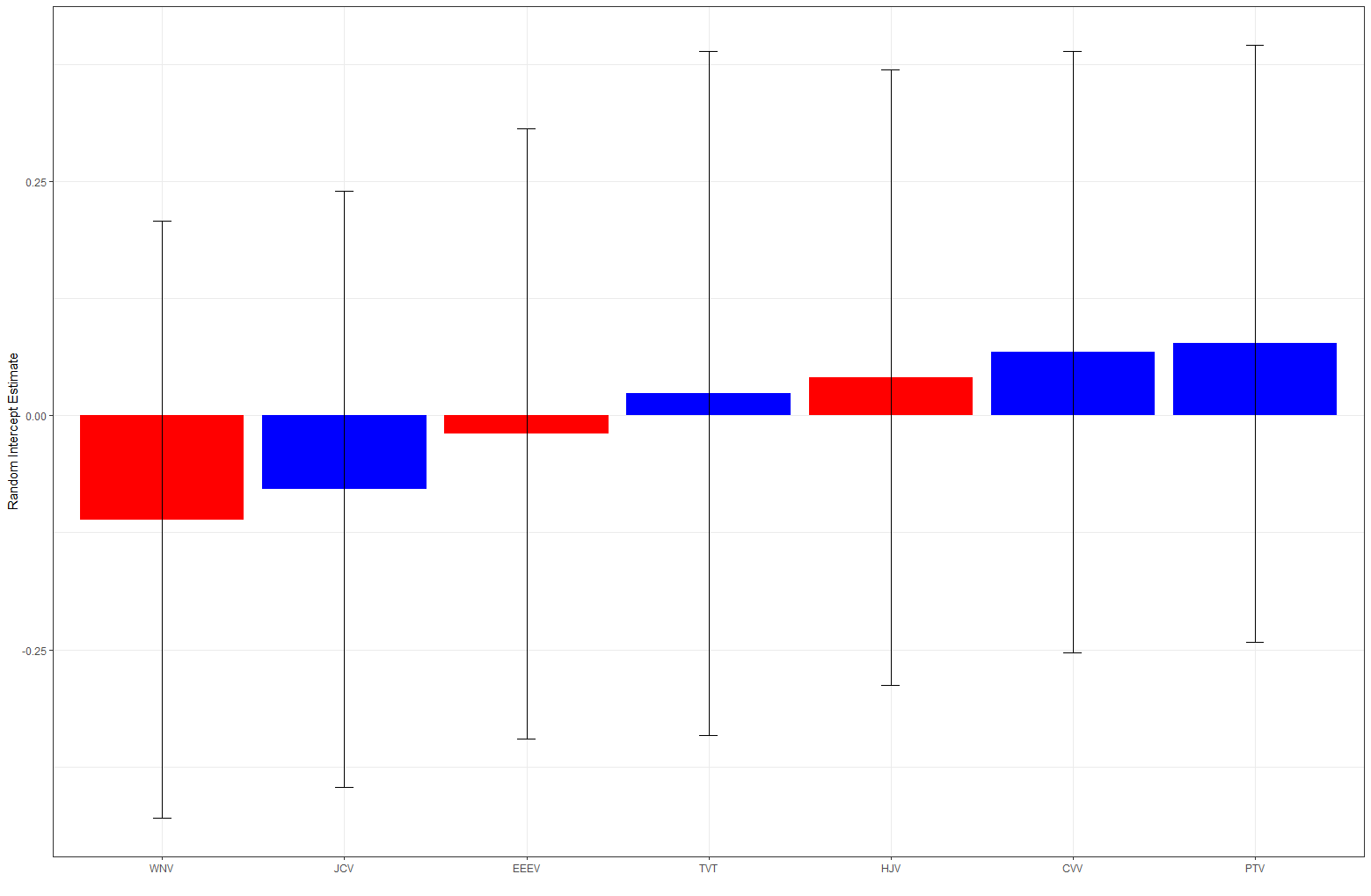


**SI Figure 3**. Arbovirus species-specific random intercept estimates of pairwise measures of time series phase synchrony for 7 arboviruses detected in CO_2_-baited, ground-level light traps set at 87 sites in Connecticut, United States from June to October from 2001 – 2020. Predictions generated from a generalized linear mixed effect model with log+1.01 transformed phase synchrony estimates, Euclidean distance, temperature similarity, habitat similarity, and a temperature/ habitat interaction as fixed effects, and arbovirus identity and site name as crossed random effects.


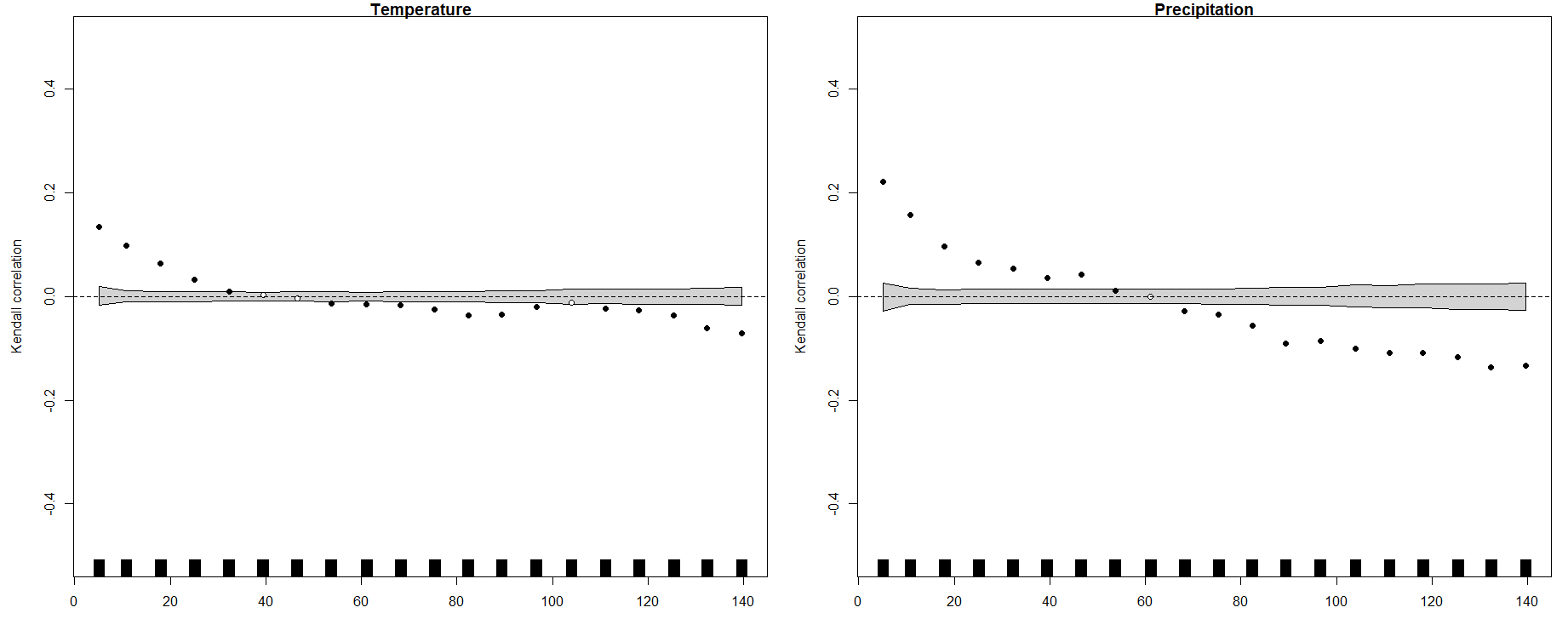


**SI Figure 4**. Spatial correlograms for temperature and precipitation (dis)similarity indices among 87 mosquito and arbovirus surveillance sites in Connecticut, U.S. Each plot is centered, meaning the regional mean is subtracted from each value. Points represent the estimated correlation per distance band (black: significant at p < 0.05; white: not significant) while the grey shaded region represents that 95%CI of the mean correlation.
